# Gangrene

**Published:** 1828-09

**Authors:** 


### HOSPITAL REPORTS,

*(Principally condensed from various Periodical Publications.)*

### GANGRENE.

Case I. *Gangrene of the Thigh, with the Question of Amputation.* By George Ballingall, m.d. (at the Royal Infirmary of Edinburgh.)

In the case of Alexander Moffat, aged seventeen, who was admitted on the 20th of May, you had an instance of a severe lacerated wound of the thigh running rapidly into gangrene, and terminating in the death of the patient. This lad's wound is described as follows:

. There is a contused wound extending from the centre of the popliteal space outwards across the knee to the fibular edge of the left patella; from near the middle of this wound there is another, which runs obliquely upwards towards the inner part of the thigh for the distance of three inches; the integuments and fascia are separated from each other all around the knee, and in many parts the latter membrane is lacerated. Two wounds of a similar character, but of trifling extent, are situated, the one in front of and a little below the inner malleolus, the other behind the tendo Achillis of the right foot. The injury was the consequence of the broad wheel of a cart pressing against his thigh for some minutes, the cart being loaded with five cwt. of marble.

On the morning of the 21st, the wound was observed to be gangrenous, and before the usual hour of visit this gangrene had spread extensively round the knee and down the forepart of the leg. Free incisions were made through the black and insensible skin, which gave vent to large quantities of fetid air and dark-coloured sanies. The propriety of amputating the limb was now considered in a full consultation, and was declined in consequence of the advanced state of the disease; the lower part of the thigh being decidedly gangrenous, and the remainder of it so far involved as to be discoloured, swollen, tender, and emphysematous. The wounds on the other leg had also assumed a gangrenous disposition; the patient was affected with subsultus tendinum, his pulse at 120 and fluttering, his tongue furred and dry, his skin hot, and thirst urgent. Hot turpentine was poured into the incisions, and the effervescing poultice applied. Opium and wine were administered internally, but without any thing like even a temporary suspension of the symptoms. He soon became delirious, and expired on the evening of the 23d.

The question which fell to be discussed in the consideration of this boy's case, the propriety of amputating during a spreading gangrene, is one which has of late occupied much of the attention of practical surgeons, and one regarding which their sentiments have recently undergone an important change. The precept of not amputating during the progress of gangrene was much too absolute to be good; and we are chiefly indebted to the military and naval surgeons of the present day for having shewn that this dogma does not admit of that indiscriminate application which was at one time given to it. In the writings of Larrey, Lawrence, Hennen, Guthrie, Hutchison, and Curtis, you will find ample encouragement and authority to deviate from what was long held to be one of the best-established rules of our art. The first of these distinguished surgeons has, by his practice and by his writings, contributed more than any other individual to establish a well-founded and practical distinction in the treatment of gangrene arising from a local, and that from a general, cause; and the last-mentioned gentleman, a naval surgeon, is particularly entitled to notice amongst those who claim the merit of priority in recommending the practice of amputating in cases of traumatic gangrene, without waiting for a line of separation; for, although his work was not published until 1807, it refers chiefly to practice instituted in the naval hospital at Madras, so far back as 1782.

Amongst other sources of information on this important point, you will find a very recent instance of the successful issue of a case in which amputation was performed during a spreading gangrene, recorded in the Edinburgh Medical and Surgical Journal, by Mr. M'Dermott, of the 4th (or King's own) Regiment, now in the castle here.

Although, for the reasons formerly stated, I declined this operation in Moffat's case, and although the only case in which I have ever operated in such circumstances terminated fatally, yet I should be sorry to have it thought that I am in any degree hostile to the practice. I think it right to observe, that, in declining an operation in the case now under review, I was in no degree influenced by the unfavorable issue of another case, which I shall immediately proceed to detail. You will recollect that at the time the amputation of Moffat's limb was under consideration, the case I allude to afforded a prospect of a favorable result. In declining the removal of this boy's limb, I was actuated by a conscientious conviction (right or wrong) of its inutility; by a firm persuasion that the performance of an operation in a case so hopeless would have been more likely to bring a promising practice into disrepute, than to have saved the life of my patient.

Case II. *Gangrene of the Leg.—Amputation.*

In the case of Robert Brockie, a patient of Mr. Liston's, you had an opportunity of seeing the limb amputated during a spreading gangrene, and, although in this instance without success, yet I do not think that, upon a fair and full consideration of the case, it can be held to argue much against a repetition of the practice. It affords another instance of those internal depositions of purulent matter succeeding to injuries or operations which have been noticed by Mr. Rose, in the last volume of the Medico-Chirurgical Transactions of London, and of which I have seen several examples. As this was a case which bears upon a great practical question, and one which very naturally and properly excited much of your attention, I am induced to give it in detail from the journals of the house.

" Robert Brockie, aged about forty, admitted 3d May, 1828; was brought in about ten p.m., having fallen from a house four stories high in Dalkeith. There is a fracture of the tibia and fibula, about an inch and a half above the ankle-joint; the lower portion of bone appears to be driven under the other. There is likewise a fracture of the second phalanx of his thumb. The limb was placed on the suspending apparatus.

8th.—The bandages round ankle-joint were yesterday removed, in consequence of pain and swelling of limb. Had some wandering, accompanied with pain of head and full pulse. Was bled to ℥xx.; blood cupped and buffed. Passed a quiet night; skin surrounding the fracture of a dusky red colour, and some vesications on fore part; pulse eighty-four, full; tongue loaded; no stool; free from pain of head.

℞. Tart. Potass. et Sodæ ℥ss.; Supertart. Potass. ℥ss.; Tart. Antimon. gr. ij.; Aquæ ℥xvj. M. capiat ℥j. tertia quaque hora.

9th.—Several large black vesications over the inner malleolus. The whole of the inside of the leg is of a dusky red colour; it is extending in a streak upwards along the inner side of the thigh; toes very cold, but is sensible when they are touched; had a good deal of starting in limb; slept badly; three natural stools; tongue moist; some thirst; skin rather hot; pulse eighty-eight, full.

A bandage applied from the toes up the thigh.—Lotio evaporat. crur.

10th.—Passed a restless night, undoing the bandages from his leg; dusky appearance has extended more towards inside of leg, and somewhat higher up the thigh; some vesications appearing on forepart of leg; no pain of head, but had some delirium last night; tongue moist; four loose stools; pulse seventy-two, full; skin cool; takes his food; foot continues cold, but feeling remains in it.

Infus. Catechu Thebai. ℥ss. subinde.—Habeat haust. h. s. c. Tinct. Opii gutt. c.

11th.—Has been sleeping soundly since one this morning; complains of no pain. Tongue much loaded; one stool; is perspiring freely; pulse 112; dusky appearance of leg much the same. It does not appear to have spread much on the thigh. The forepart of foot and toes are cold and very livid. Has taken no food this morning.

℞. Camphor. ʒss.; Emulsion. Amygdal. ℥vj. M. Habeat ℥j. secund. quaque hora.—Beef-tea ad lib.

12th.—Lies in a drowsy state, but frequently starts up in his bed. The dusky appearance on inside of thigh has entirely disappeared; the forepart of foot is more livid and cold; he does not appear to have any feeling in his toes; the discharge from ankle has a most offensive smell. No stool since yesterday; tongue loaded, much thirst; his breathing appears rather laborious; delirious at times; no pain of head; pulse 100, of good strength; skin hot. Mr. Liston removed the limb above the knee by the flap operation: there was some hemorrhage after removal to bed, in consequence of which the stump was undone, and several vessels secured. The bones were found much comminuted, the fracture extending into the ankle-joint. The cartilages were of a red appearance. There was matter of a very putrid nature running a considerable way up the calf of the leg.

13th.—Slept well; some starting of stump; has had troublesome cough this morning; breathing quite natural; pulse eighty-eight, of good strength; tongue loaded. No stool.

Beef-tea.—Contin. Mist. Camphor.

14th.—Slept well; no pain in stump; complains of pain in breast, accompanied with troublesome cough; no pain of breast on full inspiration; one scanty stool from an injection; tongue moist, but white; perspires much; pulse eighty-two; skin rather cold.

Omit. Mist. Camphor.—℞. Tinct. Digital. ʒss.; Tinct. Gentian. ℥jss. M. Capiat coc. parv. quarta quaque hora.

15th.—Slept well. Had some vomiting of bilious matter this morning; bowels not open. Had a turpentine enema, which procured one copious stool. Frequent cough; perspires much; skin cold and clammy; his whole body has a peculiar disagreeable odour; pulse fifty-five; tongue moist.

Omit. Tinct. Digital.—Habeat Spirit. Commun. ℥j. secunda quaque hora.

16th.—Slept well, but was restless during fore part of night; had some diarrhœa, on which an anodyne enema was ordered, since which he has had no stool. Takes little food; some cough; pulse fifty; skin cold and clammy; tongue clean; hiccup at times, but no vomiting. Much discharge of fetid matter from stump.

17th.—Passed rather a restless night. Has much less cough; considerable discharge from stump, which looks more healthy; pulse eighty; tongue moist and clean; less thirst; had some delirium during the night.

Habeat Tr. Opii, Camphor. ʒij. quarta quaque hora.

18th.—Passed rather a restless night. Has had at times a good deal of hiccup, but no vomiting; tongue a little loaded, but moist. One natural stool last night. Delirious during fore part of night; still a little cough; much discharge from stump of healthy looking matter; pulse seventy-two. Took some breakfast.

19th.—Had a sinapism applied to the epigastrium last night; was very restless; hiccup continues frequent. Two natural stools. Has little cough, but breathing is laborious; is perspiring much; pulse seventy-four, full; some subsultus; much discharge from stump.

℞. Spirit. Ammon. Arom. ʒij. tertia quaque hora. Rum ℥xij.

20th.—Lies in a drowsy state; no delirium. Two natural stools since yesterday. Much sweating during night; stump continues discharging; pulse ninety; no hiccup. Took some breakfast.

℞. Emulsion. Amygdal. ℥vj.; Camphor. gr. xxx. capiat ℥j. tertia quaque hora.

21st.—Passed a quiet night; hiccup returned this morning. Eat two eggs for breakfast. No stool; much sweating during night; pulse 100; much discharge from stump; countenance much improved.

22d.—Continues the same.

Two eggs for breakfast. Beef ℥vj. daily.

23d.—Complains of pain in breast, increased on full inspiration. Frequent cough, with much tenacious expectoration; slept well; one natural stool; tongue loaded; pulse ninety-six; less discharge from stump. In the space of two hours, a sudden change took place: his breathing became laborious, and at the visit he appeared rapidly sinking. He, however, rallied in the afternoon; but his breathing became again affected, and he sunk again the next morning.

25th.—On examination of body, the fourth rib was fractured about an inch from the cartilage; a small quantity of pus was found exterior to the pleura costalis; old adhesions on both sides to a great extent. The left lung was full of white tubercular bodies; several abscess in the liver; four ounces of bloody serum in pericardium. A long coagulum was found in the femoral artery.

Here, gentlemen, was a case in many circumstances the reverse of the former. The patient was more advanced in life, and evidently of a much less irritable habit; the gangrene supervened later, and was of a much less acute form: here it was situated in the extreme part of the limb, and the thigh free from swelling, tenderness, or emphysema: this, in short, was deemed by every one a fit case for experiment in a question which is perhaps still to be considered in some measure *sub judice*. One symptom, however, appeared early in this patient's case, which I did not fail to remark to my colleagues, and which, as far as my observation goes, is a circumstance almost uniformly foreboding a fatal termination: I allude to a peculiar yellow hue of the skin, which not unfrequently attends the symptomatic fever supervening upon wounds and operations. This has perhaps struck me more forcibly from being familiar with a similar appearance in the idiopathic fevers of tropical climates; and, although I have no wish to alarm the citizens of Edinburgh by talking of a yellow fever in this part of the world, yet I am bound to state, for your instruction, that I have occasionally seen it here as well marked as I ever saw it at Seringapatam or Batavia; and, when supervening upon injuries, much more uniformly fatal.

A case of this kind occurred some years ago, which made a deep impression on my mind, and which must have done so, I think, upon all those who had occasion to witness it: I allude to that of a seaman belonging to one of his Majesty's ships in the roads, whose limb had been amputated below the knee in consequence of an accident. The accommodation on board his ship being defective, and the vessel about to sail, he was brought ashore to this hospital, and placed under my care. Here his stump sloughed; the symptomatic fever ran high, was attended with that dingy yellowness of the skin to which I allude, and in a few days he died. I observed to the surgeon of the ship, who came ashore to see him dissected, that this case wanted nothing but the "black vomit" to constitute a complete example of yellow fever; and it was found, on laying open the stomach, that this circumstance, necessary to complete the parallel, was hardly wanting; for here was a large collection of that dark grumous fluid, resembling coffee grounds, which is so often evacuated from the stomach in tropical fevers.*

* From Dr. Ballingall's Clinical Lecture, July 1828.

